# An initial prediction and fine-tuning model based on improving GCN for 3D human motion prediction

**DOI:** 10.3389/fncom.2023.1145209

**Published:** 2023-04-05

**Authors:** Zhiquan He, Lujun Zhang, Hengyou Wang

**Affiliations:** ^1^Guangdong Key Laboratory of Intelligent Information Processing, Shenzhen, China; ^2^Guangdong Multimedia Information Service Engineering Technology Research Center, Shenzhen University, Shenzhen, China; ^3^School of Science, Beijing University of Civil Engineering and Architecture, Beijing, China

**Keywords:** motion prediction, GCN-based, two-stage prediction method, spatial attention, causally temporal

## Abstract

Human motion prediction is one of the fundamental studies of computer vision. Much work based on deep learning has shown impressive performance for it in recent years. However, long-term prediction and human skeletal deformation are still challenging tasks for human motion prediction. For accurate prediction, this paper proposes a GCN-based two-stage prediction method. We train a prediction model in the first stage. Using multiple cascaded spatial attention graph convolution layers (SAGCL) to extract features, the prediction model generates an initial motion sequence of future actions based on the observed pose. Since the initial pose generated in the first stage often deviates from natural human body motion, such as a motion sequence in which the length of a bone is changed. So the task of the second stage is to fine-tune the predicted pose and make it closer to natural motion. We present a fine-tuning model including multiple cascaded causally temporal-graph convolution layers (CT-GCL). We apply the spatial coordinate error of joints and bone length error as loss functions to train the fine-tuning model. We validate our model on Human3.6m and CMU-MoCap datasets. Extensive experiments show that the two-stage prediction method outperforms state-of-the-art methods. The limitations of proposed methods are discussed as well, hoping to make a breakthrough in future exploration.

## 1. Introduction

3D skeleton-based human motion prediction uses an action posture observed in the past to predict an action posture in the future. Motion prediction technology helps robots understand human behavior. This technology is of great value in areas such as intelligent security, autonomous driving (Ge et al., 2019; Djuric et al., 2020; Gao et al., 2020), object tracking, and human-robot collaboration (Liu and Wang, 2017; Oguz et al., 2017; Liu et al., 2019a, 2021; Li et al., 2020b; Liu and Liu, 2020; Ding et al., 2021; Mao et al., 2021).

Recurrent neural networks(RNN) are usually adopted to solve sequence-to-sequence prediction tasks, such as voice recognition and automatic translation (Wang et al., 2017; Tang R. et al., 2018; Iida et al., 2019; Yao et al., 2021). Due to the sequential nature of motion in the time dimension, many works use RNN to realize human motion prediction (Fragkiadaki et al., 2015; Chiu et al., 2019; Guo and Choi, 2019; Corona et al., 2020). However, RNN-based networks are usually difficult to train and have the problem of error accumulation in long-term predictions. There are a few works adopting convolution networks (CN) to solve the problem of human behavior prediction (Butepage et al., 2017; Li et al., 2018; Cui et al., 2021; Shu et al., 2021). They process the human motion sequences as images and use 2D convolution to generate the prediction sequence.

Nevertheless, human motion sequences are not traditional image data, and traditional convolution neural networks are limited in processing such sequences. In recent years, lots of work uses graph convolution networks (GCN) to solve human motion prediction tasks and achieved excellent results (Aksan et al., 2019; Cui et al., 2020; Cui and Sun, 2021; Dang et al., 2021). GCN is similar to CNN except that it performs feature extraction on graphs. GCN usually defines an adjacency matrix in advance, representing the interconnection relationship between each node in the graph. Then GCN generates new node information by aggregating related node information. In addition, by aggregating action information efficiently, recent work (Mao et al., 2019) confirms that the discrete cosine transform (DCT) has great advantages in motion prediction. Taking advantage of GCN, Many works have achieved good performance, but they also expose some shortcomings of GCN. For example, many GCN-based methods convert joint information to the frequency domain for prediction and recover the time domain information, causing the joint position generated to not smooth on the time domain. In addition, many works have changed the bone length of the human body, causing deformation.

Inspired by the proposed concept of two stages prediction (Shan et al., 2022), we present a two stages framework to solve the above problems, including the prediction stage and fine-tuning stage, to achieve precise prediction of human motion sequences. The task of the prediction stage is to use DCT to encode the motion information and then use the attention mechanism to calculate the attention score to strengthen the interaction of each node. Then we use IDCT (inverse discrete cosine transform) to decode the aggregated features into the original 3D pose, generating the initial prediction for the first stage.

We observe that the initial prediction always has a certain deviation from the ground truth. To solve this problem, we organize a fine-tuning model to correct the initial predictions of the first stage. Observing that the actors for different actions in the datasets are the same, each frame in the action sequence should contain the same body structure information, such as the length of each bone. We add a bone length constraint term in the loss function of the fine-tuning model. Since the motion sequences generated by the frequency domain are not coherent in the time domain, the traditional TCN method uses a global adjacency matrix to aggregate sequence information, which often makes predicted actions deviate from reality. In response to this problem, we propose a CMM (causal mask matrix) to improve the T-GCN and fine-tune the initial prediction, making each frame future sequence generated only related to its previous information, which eliminates the effect of future inaccurate information when constructing the current frame.

We used MPJPE as metrics to evaluate our network on the Human3.6m and CMU-MoCap, and conducted related ablation experiments to analyze our key models. Many comparative experiments show that our method achieves more accurate predictions than the existing approaches.

In summary, the main contributions of this paper can be concluded as follows:

- We propose a two-stage training method, including prediction and fine-tuning stages. Fine-tuning stage corrects the human motion sequences generated by the prediction stage.
- To further utilize the interactive information on the temporal structure of human motion, we present a CMM improving the T-GCN in the fine-tuning stage to reconstruct the sequence in a causal, temporal order.
- In order to improve the power of GCN to extract the spatial interaction information of the human, we introduce a SAB (spatially attention block) to aggregate node information along the spatial dimension. Moreover, we incorporate the constraint of length invariance of human bones for guiding the framework to generate more realistic human motion sequences.

## 2. Related work

### 2.1. RNN-based method

RNN-based methods are widely used for sequence-to-sequence tasks (Jain et al., 2016b; Martinez et al., 2017; Tang Y. et al., 2018; Liu et al., 2019b; Sang et al., 2020). According to the characteristics of human motion sequence, a lot of works use RNN as the basic structure of the network. By embedding encoder and decoder networks before and after recurrent layers, Fragkiadaki et al. (2015) propose an Encoder-Recurrent-Decoder (ERD) model for predicting human motion. Jain et al. (2016a) combine RNNs with the spatiotemporal structure of the human body, proposing the Structural-RNN. Liu et al. (2019b) develop a hierarchical recurrent network structure to simultaneously encode the local context of a single frame and the global context of a sequence.

However, RNN combines the hidden layer of the previous unit to output the prediction of the next unit, which will cause the accumulation of errors. These methods cannot avoid the error accumulation problem. Error accumulation causes discontinuities in generated frames, resulting in unrealistic human motion sequences. Gui et al. (2018) propose a novel sequence-to-sequence model that adds a residual architecture connection between the input and output of each RNN module, which alleviates the discontinuity problem of the RNN model. Guo and Choi (2019) modified the seq2seq framework to encode temporal correlations at different time scales. Shu et al. (2021) designed a new bone- joint attention mechanism to dynamically learn the bone-joint feature map of the bone-joint attention feature map, making the generated action sequences closer to reality. Although these methods effectively improve the accuracy of prediction, their performance in long-term prediction is still insufficient.

### 2.2. GCN-based method

Compared with traditional CNN-based methods, the GCN-based method has significant advantages in the face of irregular data structures, such as social networks (Tabassum et al., 2018; Li et al., 2020a) and human body posture and behavior (Fan et al., 2019; Chen et al., 2020). In recent years, graph neural networks have been widely used for 3D human motion prediction and have achieved outstanding results. Lebailly et al. (2020) use GCN as an encoder and GCN to decode the aggregated features. The works of Mao et al. (2019), Cui et al. (2020), and Dang et al. (2021) totally used GCN to organize the model. Li et al. (2020b) encode the human body into multiple scales and perform information fusion, proposing the DMGNN. Ma et al. (2022) used a spatiotemporal GCN to build a model to obtain more accurate long-term predictions by predicting the median value of human motion. Mao et al. (2019) used discrete cosine transform to encode human motion sequences and designed a GCN-based model that automatically learns node relationships.

Although these GCN-based methods have achieved good results, these methods still have not solved the following problems: The method predicts in the frequency domain often cannot pay attention to the time dependence of the original information. The future human motion sequence generated by these methods does not follow the bone constraints. In other words, the length of the human bone skeletal generated has changed. In order to solve the time dependence of frequency domain prediction methods, we propose a two-stage network architecture. The first stage is initially predicted in the frequency domain, and the second stage fine-tunes the initial result in the time domain. In the second stage, we use CMM to change the adjacent matrix into causality. In order to make the prediction results follow the bone constraints, we proposed the SAB to enhance the ability to capture the Spatial interaction relationship of the joint and increase all bone length as a constraint to train the model. We introduce the details of our framework architecture in the following section.

## 3. Problem formulation

Suppose that *X*_−*T*_*p*_:0_ = [*X*_−*T*_*p*__, …, *X*_0_] denotes the historical human motion sequence of length *T*_*p*_ + 1 and *X*_1:*T*_*f*__ = [*X*_1_, …, *X*_*T*_*f*__] denotes the future sequence of length *T*_*f*_, where $X_{i}\in {\mathbb{R}}^{N\times D}$ with *N* joints and *D* = 3 feature- dimensions depicts the 3D human pose at time *i*. The task of 3D human motion prediction is to generate the future sequence *X*_1:*T*_*f*__ given the historical one.

For predicting complex human motion more accurately, we use a two-stage prediction method based on GCN. We use cascading SAGCLs to predict the results of the first stage. Then, the initial prediction is fine-tuned by using the space-time constraints of the human body to get the second stage prediction that is closer to real human movement.

## 4. Methodology

### 4.1. Prediction and fine-tuning framework

**Figure 1:**
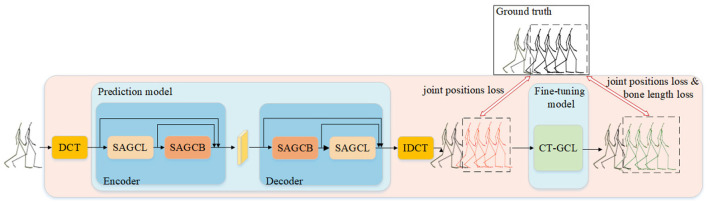
Overview of our prediction and fine-tuning human motion prediction framework containing a prediction model and a fine-tuning model. SAGCL denotes the spatial attention graph convolution layer, and the SAGCB consists of cascade SAGCL. Black skeleton represents group truth. The red skeleton represents the prediction of the first stage, and the green skeleton represents the result of fine-tuning.

In order to predict future motion sequences precisely, we adopt a two-stage training method, as shown in Figure 1. According to the human motion of T frames observed in the past *X*_−_ = [*X*_−*T*_, *X*_−*T*+1_, …, *X*_−1_], we first apply DCT along the time dimension to convert the temporal dynamics of motions into the frequency domain.

The encoder in the predicted model uses the node information in the frequency domain of SAGCL and SAGCB (Spatial Attention Graph Convolution Block), and then generates the predictive information of the frequency domain through the same structured decoder. Then the prediction model will use IDCT to restore the predicted joint information to the time domain. As shown in Figure 1, the red skeleton in the middle denotes the initial prediction of the first stage. We use the joint position errors as constraints to train the prediction model. Meanwhile, the skeleton is marked as red, representing the problem of discontinuity and skeletal deformation.

We reduce the impact of these problems in the second stage. CT-GCL only predicts depending on the past sequence. And we consider the joint position and the bone length constraint to train the fine-tuning model. The second stage corrects the bone length and keeps temporal dependence, which makes the prediction closer to natural human motion.

### 4.2. Prediction model (frequency domain)

**Figure 2:**
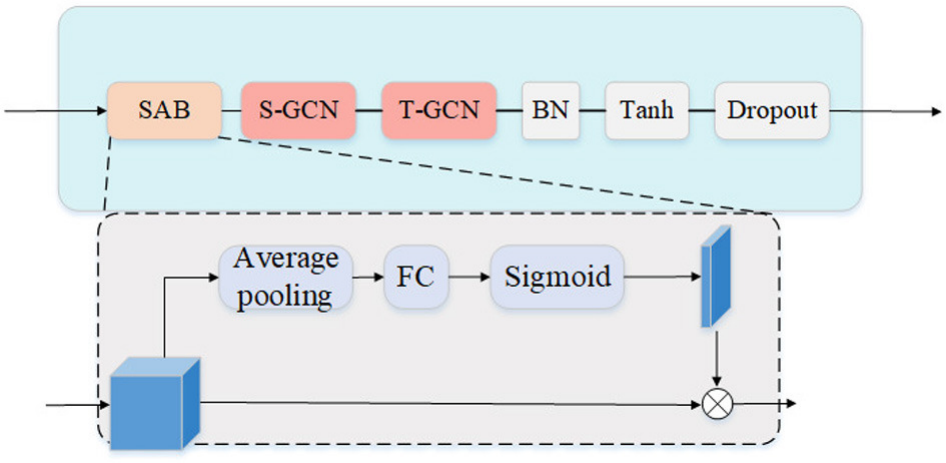
The structure of SAGCL. SAB denotes spatial attention block. S-GCN and T-GCN indicates the Spatial and temporal GCN, respectively. BN indicates batch normalize operation.

Based on S-GCN (Spatial-Graph Convolution Layer) and T-GCN (temporal-Graph Convolution Layer), we build an encoder-decoder human motion prediction model. Both the encoder and decoder contain a SAGCL and a SAGCB. Each SAGCB includes six SAGCLs. The structure of SAGCL is shown in Figure 2. When the motion information flows through SAGCL, SAB will first extract the interaction information between human joints based on the motion information. In SAB, we use the average pooling layer to aggregate human body node interactive information along the spatial dimension. SAB can calculate the gating weight value of each node in the interval 0 − 1 according to the Sigmoid function and finally aggregate the information between dependent nodes according to the dynamic joint weight. The weight matrix of SAB can be calculated by the following formula:


(1) $$A_{SAB}=\text{Sigmoid}(W\text{AvgPool}(H)+b),$$

Where *H* represents the hidden feature. **W** and **b** are the Parameter matrix and bias vector of FC layer, respectively. AvgPool denotes the average pooling along the temporal dimension. The Sigmoid function calculates the jointswise 0 − 1 gating weights.

Then S-GCN aggregates interaction information along the spatial dimensions. Let *X*∈*R*^*L*×*M*×*F*^ be a pose sequence where L is the length of the sequence, M is the number of joints of a pose, and F indicates the number of features of a joint. Defining a learnable adjacency matrix $A_{s}\in R^{M\times M}$ the elements of which measure relationships between pairs of joints of a pose, S-GCN work as:


(2) $$\begin{array}{l} X^{l+1}=\sigma \left(A_{SAB}A_{s}X^{l}W_{s}^{l}\right), \end{array}$$

Where *l* denotes the parameter in *l*^*th*^ layer. σ represents the Leaky ReLU. T-GCN aggregates interaction information along the temporal dimensions. Defining a learnable adjacency matrix $A_{t}\in R^{L\times L}$ measuring weights between pairs of joints of a trajectory, T-GCN computes:


(3) $$\begin{array}{l} Y^{l+1}=\sigma \left(A_{t}Y^{l}W_{t}^{l}\right). \end{array}$$

### 4.3. Fine-tuning model (time domain)

**Figure 3:**
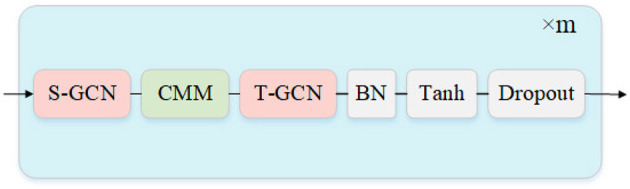
The structure of fine-tuning model. CMM denotes causal mask matrix that improve the T-GCN. The hyperparameter m denotes the cascade number of “CT-GCL”.

Previous work based on graph convolution often focuses on global historical information through the temporal adjacency matrix when generating future action sequences. But the results predicted by the frequency domain are only sometimes smooth in time series. Using the unsmoothed global history motion information tends to corrupt the future sequence generated by the network. Therefore, we build a Fine-tuning model based on a cascade CT-GCL to reconstruct all the future sequences in the time domain. The input of the fine-tuning model is the complete output of the prediction model. The output of the fine-tuning model is a new sequence adjusted by constraints. As shown in Figure 3, each CT-GCL is mainly established by the S-GCN, CMM, and T-GCN, creating a new mapping between the temporal independent motion sequence and the temporal causal sequence.

**Figure 4:**
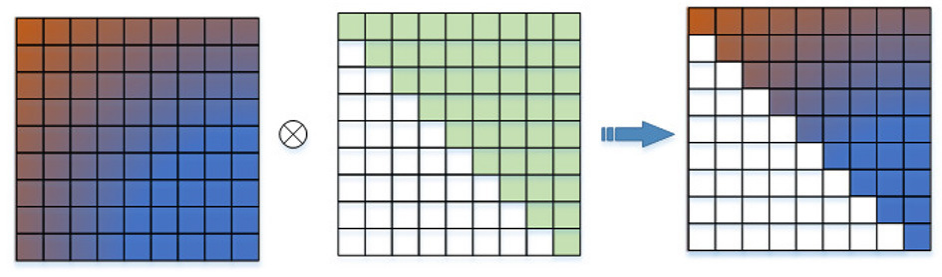
The temporal adjacency matrix makes hadamard product with CMM.

CMM adjusts the node position predicted in the first stage in the temporal series so that the node position at each moment is only related to the previous time. As shown in Figure 4, CMM is initialized as an upper triangular matrix and makes a Hadamard product with the adjacency matrix in the temporal dimension. So only the information at the current moment and before is aggregated when CT-GCL reconstructs the future motion sequence.

### 4.4. Loss function

For training the prediction network, we consider *L*_2_ loss function for 3D joint positions. Suppose that the prediction sample is $\hat{\chi }$, and the corresponding ground truth value is χ. For T training samples and K nodes, the loss function is:


(4) $$\begin{array}{l} L_{\text{prediction}}\left(\chi ,\hat{\chi }\right)=\frac{1}{KT}\overset{K}{\underset{k=1}{\sum }}\overset{T}{\underset{t=1}{\sum }}||p_{t}^{k}-{\hat{p}}_{t}^{k}|{|}_{2}, \end{array}$$

Where $p_{t}^{k}$ denotes the ground truth position of k-th joint in frame t and ${\hat{p}}_{t}^{k}$ denotes the predicted one. We adopts *L*2 loss function for 3D joint positions and the length of each bone in a human body to train the fine-tuning network. The loss function is:


(5) $$\begin{array}{l} \begin{array}{c} L_{\text{fine-tuning}}\left(\chi ,\hat{\chi }\right)=\frac{1}{KT}\overset{K}{\underset{k=1}{\sum }}\overset{T}{\underset{t=1}{\sum }}||p_{t}^{k}-{\hat{p}}_{t}^{k}|{|}_{2}\\ +\frac{1}{MT}\overset{M}{\underset{m=1}{\sum }}\overset{T}{\underset{t=1}{\sum }}||b_{t}^{m}-{\hat{b}}_{t}^{m}|{|}_{2}, \end{array} \end{array}$$

Where $b_{t}^{m}$ denotes the length of the *M*^*th*^ bone in the human body, and ${\hat{b}}_{t}^{m}$ denotes the length of the *M*^*th*^ bones in frame t. $p_{t}^{k}$ and ${\hat{p}}_{t}^{k}$ are the same as in formula (4). By correcting the length of the bones in each frame of the predicted sequence, the fine-tuning network makes the reconstructing sequence closer to the actual value.

## 5. Experiments

We used Human 3.6m (Ionescu et al., 2013) and CMU-MoCap dataset to validate our framework. The joint data for both datasets are represented by an exponential map. In this work, We convert it to a 3D coordinate representation. Furthermore, we show the quantitative results for both short-term and long-term human motion predictions for joint positions by Mean Per Joint Position Error (MPJPE).

### 5.1. Datasets

#### 5.1.1. CMU−MoCap

CMU-MoCap has 5 main categories of motion data. In line with previous work (Mao et al., 2019; Dang et al., 2021), we selected eight actions to validate our framework: “basketball”, “basketball signal”, “directing traffic”, “jumping”, “running”, “soccer”, “walking”, and “washing window”. Each motion data contains 38 joints (contains repeated joints), and we preserve 25 valuable joints. The division of the training set and test set also remains the same as Mao et al. (2019) and Dang et al. (2021).

#### 5.1.2. Human3.6m

Human3.6m has 15 different classes of motion performed by 7 actors. Each motion in subjects contains 32 joints, and we preserve 22 joints. To be consistent with Li et al. (2020b), we train the model on 6 subjects and test it on the 5th subject. To be consistent with previous work (Dang et al., 2021), we use S1, S6, S7, S8, and S9 for training and use S5 and S11 for testing and validation, respectively.

### 5.2. Metrics

In this paper, we train and test the 3D coordinates coordinate representation of the human pose and show the measurement results in 3D coordinates. Defining the prediction sample is $\hat{X}$, and the corresponding ground truth value is X. We use mean per-joint position error (MPJPE) as an evaluation metric for 3D error:


(6) $$\begin{array}{l} \mathrm{MPJPE}\left(X,\hat{X}\right)=\frac{1}{NT}\overset{N}{\underset{n=1}{\sum }}\overset{T}{\underset{t=1}{\sum }}||p_{t}^{n}-{\hat{p}}_{t}^{n}|{|}_{2}, \end{array}$$

Where $p_{t}^{n}$ represents the *n*^*th*^ ground truth joint position in *t*^*th*^ frame. And ${\hat{p}}_{t}^{n}$ denotes the predictive one.

### 5.3. Model configuration

There are different cross-validation methods, such as k-fold cross-validation and jack-knife test, which have been generally used to train the model (Arif et al., 2021; Ge et al., 2021, 2022a,b; Sikander et al., 2022). We trained our proposed model using a 10-fold cross-validation method. Our network predicts the human pose of 25 frames in the future by observing the position of the joints in the past 10 frames. Each SAGCB in the prediction model contains 6 SAGCLs. After testing, we cascaded 6 CT-GCLs in the fine-tuned model. We utilize Adam as an optimizer. The learning rate is initialized to 0.005 with a 0.96 decay every epoch. Both the prediction model and the fine-tuning model are trained for 50 epochs, and the batch size is set to 32. We implemented our network on GeForce RTX 2080 Ti GPU using Pytorch.

### 5.4. Comparison to state-of-the-art methods

We validated our model on Human3.6m and CMU-MoCap datasets and present detailed results. We compared our method with DMGNN (Li et al., 2020b), LTD (Mao et al., 2019), MSR (Dang et al., 2021), and ST-DGCN (Ma et al., 2022). DMGNN uses GCN to extract features of multiple scales of the human body and uses graph-based GRU for decoding. Applying DCT, LTD uses GCN for prediction in the frequency domain. MSR is improved on the basis of LTD, taking into account multi-scale factors of the human body.

#### 5.4.1. Human3.6m

As seen in Table 1, we compared the several methods mentioned above in short-term prediction (within 400 ms) on Human3.6m. The results show that our method outperforms previous methods in short-term prediction. For example, our method has a significant advantage in the action “greeting”, “posing”, and “sit down”. Our method is greatly reduced in the short -term prediction of many actions such as “walkingdog”, “greeting”, and “discussion”, which performance is more than 10% higher than the best method in 80 ms. And Table 2 shows the comparisons of long-term prediction (between 400 and 1,000 ms). In most cases, our results are better than the compared methods. For example, the performance of our method in motion “discussion” and “posing” is about 3% higher than the best method in 1,000 ms and motion “walkingtogether” is more than 5% higher, which shows that our method also performs well in the longest prediction. According to the average errors for short-term and long-term prediction, our method outperforms the compared methods by a large margin.

**Table 1:** Comparisons of short-term prediction on Human3.6m. Results at 80, 160, 320, 400 ms in the future are shown.

**Scenarios**	**Walking**				**Eating**				**Smoking**				**Discussion**			

**Millisecond**	**80 ms**	**160 ms**	**320 ms**	**400 ms**	**80 ms**	**160 ms**	**320 ms**	**400 ms**	**80 ms**	**160 ms**	**320 ms**	**400 ms**	**80 ms**	**160 ms**	**320 ms**	**400 ms**
DMGNN	17.3	30.7	54.6	65.2	11.0	21.4	36.2	43.9	9.0	17.6	32.1	40.3	17.3	34.8	61.0	69.8
LTD	12.3	23.0	39.8	46.1	8.4	16.9	33.2	40.7	7.9	16.2	31.9	38.9	12.5	27.4	58.5	71.7
MSR	12.2	22.7	38.6	45.2	8.4	17.1	33.0	40.4	8.0	16.3	31.3	38.2	12.0	26.8	57.1	69.7
PGB	10.2	19.8	**34.5**	**40.3**	7.0	15.1	30.6	38.1	6.6	14.1	28.2	**34.7**	10.0	23.8	53.6	66.7
Ours	**9.5**	**19.0**	34.8	41.4	**6.6**	**14.5**	**30.1**	**37.7**	**6.2**	**13.6**	**27.9**	34.8	**9.3**	**22.8**	**52.7**	**66.0**
**Scenarios**	**Directions**				**Greeting**				**Phoning**				**Posing**			
**Millisecond**	**80 ms**	**160 ms**	**320 ms**	**400 ms**	**80 ms**	**160 ms**	**320 ms**	**400 ms**	**80 ms**	**160 ms**	**320 ms**	**400 ms**	**80 ms**	**160 ms**	**320 ms**	**400 ms**
DMGNN	13.1	24.6	64.7	81.9	23.3	50.3	107.3	132.1	12.5	25.8	48.1	58.3	15.3	29.3	71.5	96.7
LTD	9.0	19.9	43.4	53.7	18.7	38.7	77.7	93.4	10.2	21.0	42.5	52.3	13.7	29.9	66.6	84.1
MSR	8.6	19.7	43.3	53.8	16.5	37.0	77.3	93.4	10.1	20.7	41.5	51.3	12.8	29.4	67.0	85.0
PGB	7.2	17.6	40.9	**51.5**	15.2	34.1	71.6	87.1	8.3	18.3	38.7	48.4	10.7	25.7	60.0	76.6
Ours	**6.7**	**17.0**	**40.6**	**51.5**	**13.8**	**31.9**	**69.2**	**85.8**	**7.8**	**17.7**	**38.2**	**48.3**	**9.3**	**23.7**	**57.3**	**73.8**
**Scenarios**	**Purchases**				**Sitting**				**Sittingdown**				**Takingphoto**			
**Millisecond**	**80 ms**	**160 ms**	**320 ms**	**400 ms**	**80 ms**	**160 ms**	**320 ms**	**400 ms**	**80 ms**	**160 ms**	**320 ms**	**400 ms**	**80 ms**	**160 ms**	**320 ms**	**400 ms**
DMGNN	21.4	38.7	75.7	92.7	11.9	25.1	44.6	**50.2**	15.0	32.9	77.1	93.0	13.6	29.0	46.0	58.8
LTD	15.6	32.8	65.7	79.3	10.6	21.9	46.3	57.9	16.1	31.1	61.5	75.5	9.9	20.9	45.0	56.6
MSR	14.8	32.4	66.1	79.6	10.5	22.0	46.3	57.8	16.1	31.6	62.5	76.8	9.9	21.0	44.6	56.3
PGB	12.5	28.7	60.1	**73.3**	8.8	19.2	**42.4**	53.8	13.9	27.9	57.4	71.5	8.4	18.9	42.0	**53.3**
Ours	**11.8**	**27.8**	**60.0**	73.9	**8.3**	**18.2**	41.1	52.5	**13.0**	**26.4**	**55.2**	**69.4**	**8.0**	**18.3**	**41.6**	**53.0**
**Scenarios**	**Waiting**				**Walkingdog**				**Walkingtogether**				**Average**			
**Millisecond**	**80 ms**	**160 ms**	**320 ms**	**400 ms**	**80 ms**	**160 ms**	**320 ms**	**400 ms**	**80 ms**	**160 ms**	**320 ms**	**400 ms**	**80 ms**	**160 ms**	**320 ms**	**400 ms**
DMGNN	12.2	24.2	59.6	77.5	47.1	93.3	160.1	171.2	14.3	26.7	50.1	63.2	17.0	33.6	65.9	79.7
LTD	11.4	24.0	50.1	61.5	23.4	46.2	83.5	96.0	10.5	21.0	38.5	45.2	12.7	26.1	52.3	63.5
MSR	10.7	23.1	48.3	59.2	20.7	42.9	80.4	93.3	10.6	20.9	37.4	43.9	12.1	25.6	51.6	62.9
PGB	8.9	20.1	43.6	54.3	18.8	39.3	73.7	86.4	8.7	18.6	**34.4**	**41.0**	10.3	22.7	47.4	58.5
Ours	**8.4**	**19.3**	**42.8**	**54.0**	**16.9**	**36.6**	**71.2**	**84.7**	**8.4**	**18.2**	**35.2**	**42.3**	**9.6**	**21.7**	**46.5**	**57.9**

The best results are highlighted in bold.

**Table 2:** Comparisons of long-term prediction on Human3.6M.

**Scenarios**	**Walking**		**Eating**		**Smoking**		**Discussion**		**Directions**		**Greeting**		**Phoning**		**Posing**	
**Millisecond**	**560 ms**	**1,000 ms**	**560 ms**	**1,000 ms**	**560 ms**	**1,000 ms**	**560 ms**	**1,000 ms**	**560 ms**	**1,000 ms**	**560 ms**	**1,000 ms**	**560 ms**	**1,000 ms**	**560 ms**	**1,000 ms**
DMGNN	73.4	95.8	58.1	86.7	50.9	72.2	81.9	138.3	110.1	115.8	152.5	157.7	78.9	**98.6**	163.9	310.1
LTD	54.1	59.8	53.4	77.8	50.7	72.6	91.6	121.5	71.0	101.8	115.4	148.8	69.2	103.1	114.5	173.0
MSR	52.7	63.0	52.5	77.1	49.5	71.6	88.6	117.6	71.2	100.6	116.3	147.2	68.3	104.4	116.3	174.3
PGB	**48.1**	56.4	51.1	76.0	46.5	69.5	**87.1**	118.2	**69.3**	**100.4**	**110.2**	143.5	**65.9**	102.7	**106.1**	164.8
Ours	48.7	**56.1**	**50.7**	**73.4**	**46.1**	**68.5**	**87.1**	**115.6**	70.7	101.0	111.4	**142.2**	66.9	101.6	107.3	**161.7**
**Scenarios**	**Purchases**		**Sitting**		**Sittingdown**		**Takingphoto**		**Waiting**		**Walkingdog**		**Walkingtogether**		**Average**	
**Millisecond**	**560 ms**	**1,000 ms**	**560 ms**	**1,000 ms**	**560 ms**	**1,000 ms**	**560 ms**	**1,000 ms**	**560 ms**	**1,000 ms**	**560 ms**	**1,000 ms**	**560 ms**	**1,000 ms**	**560 ms**	**1,000 ms**
DMGNN	118.6	153.8	**60.1**	**104.9**	122.1	168.8	91.6	120.7	106.0	136.7	194.0	182.3	83.4	115.9	103.0	137.2
LTD	102.0	143.5	78.3	119.7	100.0	150.2	77.4	119.8	79.4	108.1	111.9	148.9	55.0	65.6	81.6	114.3
MSR	101.6	139.2	78.2	120.0	102.8	155.5	77.9	121.9	76.3	106.3	111.9	148.2	52.9	65.9	81.1	114.2
PGB	**95.3**	**133.3**	74.4	116.1	96.7	147.8	74.3	118.6	**72.2**	**103.4**	104.7	139.8	51.9	64.3	**76.9**	110.3
Ours	95.5	135.9	74.4	116.2	**96.1**	**147.3**	**74.1**	**117.3**	73.7	104.8	**104.5**	**138.5**	**50.7**	**61.0**	77.2	**109.4**

The best results are highlighted in bold.

#### 5.4.2. CMU−MoCap

Table 3 shows the comparisons of average value on CMU-MoCap. Our method significantly outperforms the comparison methods in both short-term and long-term prediction. The error of our method is reduced by nearly 10% compared with ST-DGCN in 1,000 ms prediction.

**Table 3:** Comparisons of average prediction errors on CMU-MoCap in both short-term and long-term prediction.

**Millisecond**	**80 ms**	**160 ms**	**320 ms**	**400 ms**	**560 ms**	**1,000 ms**
DMGNN	13.6	24.1	47.0	58.8	77.4	112.6
LTD	9.3	17.1	33.0	40.9	55.8	86.2
MSR	8.1	15.2	30.6	38.6	53.7	83.0
PGB	7.6	14.3	29.0	36.6	50.9	80.1
Ours	**7.1**	**13.2**	**26.8**	**34.1**	**47.6**	**72.4**

The best results are highlighted in bold.

### 5.5. Ablation study

To further analyze our model, we performed the following ablation studies on Human3.6m. We conduct the following comparative experiments to analyze the impact of each module of our model.

As shown in Table 4, we tested the performance of the predictive model alone using the ground truth to evaluate the effect of the fine-tuned model. In the case of only the forecasting model, both long-term and short-term predictions have performance degradation. The average prediction error went from 53.71 to 55.08. Experiments show that the fine-tuning module adjusts the initial prediction by time dependence and bone constraint, making the predicted motion sequence closer to the actual value. We also tested the impact of several key modules in the framework, such as SAB, CMM, and bone length loss function. The average error of melting the above key modules has risen to 54.31, 54.13, and 54.53, respectively.

**Table 4:** Ablations on architecture.

	**80 ms**	**160 ms**	**320 ms**	**400 ms**	**560 ms**	**1,000 ms**	**Average**
Only prediction model	10.8	23.7	48.4	59.4	77.6	110.6	55.08
Without SAB	10.2	22.3	47.5	58.3	77.5	110.1	54.31
Without CMM	9.9	22	47.7	58.5	**77.2**	109.5	54.13
Without bone length loss function	10.4	22.6	47.9	58.8	77.3	110.2	54.53
Full model	**9.6**	**21.7**	**46.5**	**57.9**	**77.2**	**109.4**	**53.71**

We check the output of the prediction model only. Compare the full model and the full model without the key model we presented such as SAB, CMM, and the bone length constraint. The best results are highlighted in bold.

Our results show that the bone length loss function has the most significant impact on the model, which verifies the problem of the GCN-based method in predicting the deformation of the human body. Our method uses SAB and bone constraints to strengthen the extraction of bone information by GCN layers, making our results better than the current method. The CMM module has also played a positive role in fine-tuning modules to avoid discontinuous information in the future to destroy the aggregation of temporal information in T-GCN.

As shown in Table 5, keeping the output of the prediction model constant, we conduct ablations about the cascaded layers m from 4 to 7 in the fine-tuning model. The results show that the fine-tuned model has the best performance when *m* = 6.

**Table 5:** Results comparsion of different number m of “CT-GCL” cascade, which mentioned in Figure 3.

	**80 ms**	**160 ms**	**320 ms**	**400 ms**	**560 ms**	**1,000 ms**	**Average**
*m* = 4	10.1	22.1	47	58.8	77.6	110.2	54.30
*m* = 5	**9.5**	**21.6**	46.6	58.2	77.4	109.7	53.83
*m* = 6	9.6	21.7	**46.5**	**57.9**	**77.2**	**109.4**	**53.71**
*m* = 7	9.8	21.8	46.7	58.3	77.4	109.6	53.93

After the experiment, we finally set the test range at 4–7. The best results are highlighted in bold.

Table 6 shows the comparison of different numbers of SAGCLs in a SAGCB. We regard the prediction model and fine-tuning model as two independent modules. And we only consider the results of the prediction model in this experiment. The results showed that five SAGCLs achieved the most accurate results at 80 ms. However, in the long-term error comparison, six SAGCLs have more advantages. Considering the average error, we use six SAGCLs to obtain the most accurate initial prediction.

**Table 6:** Results comparsion of different numbers of SAGCLs in a SAGCB.

**SAGCLs**	**80 ms**	**160 ms**	**320 ms**	**400 ms**	**560 ms**	**1,000 ms**	**Average**
4	10.7	**23.7**	48.8	60.2	78.5	111.7	55.60
5	**10.5**	**23.7**	**48.4**	59.8	77.9	110.8	55.18
6	10.8	**23.7**	**48.4**	**59.4**	**77.6**	**110.6**	**55.08**
7	10.9	23.9	48.6	60.0	78.1	111.2	55.45
8	11.3	24.3	49.2	60.8	78.7	112.0	56.05

After the experiment, we finally set the test range at 4 to 8. The best results are highlighted in bold.

## 6. Discussion

In the previous section, we compared our method with state of the art. Using SAB and bone length constraint, our method has a strong spatial interactive relationship capture ability. Thus, our method has a significant advantage in some motions with large movements, such as a result of action “walking dogs” and “sitting down” in Tables 1, 2. As shown in Table 3, using a fine-tuning model to adjust the initial prediction in the time domain. Our model has a strong ability to capture time dependence so that the performance of the model is more than 10% compared with the latest method by 1,000 ms. As shown in Table 4, CMM also enhances this performance.

On the other hand, there are also some shortcomings in our model. Our model is based on the two-stage training method. We need to pre-training a prediction model and then train a fine-tuning model, which undoubtedly increases our training time and complexity. What is more, our fine-tuning model is largely limited by predictive models, which means that the correction capacity of fine-tuning models is limited. We still do not do well in long time prediction. As shown in Table 2, we still have a lot of room for improvement in long-term predictions.

## 7. Conclusion

We propose a two-stage forecasting framework, including prediction and fine-tuning models. In the prediction model, we first transform the observed pose data into the frequency domain using DCT. Before the transformed pose data flows through the GCN, the interaction information between joints is enhanced by the spatial attention mechanism. Then we use IDCT to restore the generated future poses to the time domain. In the second stage, we add the bone length error as a loss function to train the fine-tuning model better, which makes the corrected pose sequence closer to the natural human motion. What is more, we use CMM to improve the T-GCN in the fine-tuning model, making the regenerated motion sequences more coherent on the timeline. Extensive experiments show that fine-tuning the model plays a positive role in improving the results of the predictive model. Our work outperforms previous work on commonly used datasets.

## Data availability statement

Publicly available datasets were analyzed in this study. This data can be found here: http://mocap.cs.cmu.edu/.

## Author contributions

LZ proposed the two stage training method for human motion production and wrote the manuscript. ZH conducted the literature survey and method guidance. HW analyzed the experiment data and revised the manuscript.

## References

[B1] Aksan E. Kaufmann M. Hilliges O. (2019). “Structured prediction helps 3D human motion modelling,” in Proceedings of the IEEE/CVF International Conference on Computer Vision (Seoul: IEEE), 7144–7153.

[B2] Arif M. Kabir M. Ahmed S. Khan A. Ge F. Khelifi A.. (2021). Deepcppred: a deep learning framework for the discrimination of cell-penetrating peptides and their uptake efficiencies. IEEE/ACM Trans. Comput. Biol. Bioinform. 19, 2749–2759. 10.1109/TCBB.2021.3102133 34347603

[B3] Butepage J. Black M. J. Kragic D. Kjellstrom H. (2017). “Deep representation learning for human motion prediction and classification,” in Proceedings of the IEEE Conference on Computer Vision and Pattern Recognition (Honolulu, HI: IEEE), 6158–6166.

[B4] Chen G. Song X. Zeng H. Jiang S. (2020). Scene recognition with prototype-agnostic scene layout. IEEE Trans. Image Process. 29, 5877–5888. 10.1109/TIP.2020.2986599 32305915

[B5] Chiu H.-K. Adeli E. Wang B. Huang D.-A. Niebles J. C. (2019). “Action-agnostic human pose forecasting,” in 2019 IEEE Winter Conference on Applications of Computer Vision (WACV) (Waikoloa, HI: IEEE), 1423–1432.

[B6] Corona E. Pumarola A. Alenya G. Moreno-Noguer F. (2020). “Context-aware human motion prediction,” in Proceedings of the IEEE/CVF Conference on Computer Vision and Pattern Recognition (Seattle, WA: IEEE), 6992–7001.

[B7] Cui Q. Sun H. (2021). “Towards accurate 3D human motion prediction from incomplete observations,” in Proceedings of the IEEE/CVF Conference on Computer Vision and Pattern Recognition, 4801–4810.

[B8] Cui Q. Sun H. Kong Y. Zhang X. Li Y. (2021). Efficient human motion prediction using temporal convolutional generative adversarial network. Inform. Sci. 545, 427–447. 10.1016/j.ins.2020.08.123

[B9] Cui Q. Sun H. Yang F. (2020). “Learning dynamic relationships for 3D human motion prediction,” in Proceedings of the IEEE/CVF Conference on Computer Vision and Pattern Recognition (Honolulu, HI: IEEE), 6519–6527.

[B10] Dang L. Nie Y. Long C. Zhang Q. Li G. (2021). “MSR-GCN: multi-scale residual graph convolution networks for human motion prediction,” in Proceedings of the IEEE/CVF International Conference on Computer Vision (IEEE), 11467–11476.

[B11] Ding Z. Yang C. Wang Z. Yin X. Jiang F. (2021). Online adaptive prediction of human motion intention based on sEMG. Sensors 21, 2882. 10.3390/s21082882 33924152 PMC8074390

[B12] Djuric N. Radosavljevic V. Cui H. Nguyen T. Chou F.-C. Lin T.-H.. (2020). “Uncertainty-aware short-term motion prediction of traffic actors for autonomous driving,” in Proceedings of the IEEE/CVF Winter Conference on Applications of Computer Vision (Snowmass, CO: IEEE), 2095–2104.

[B13] Fan L. Wang W. Huang S. Tang X. Zhu S.-C. (2019). “Understanding human gaze communication by spatio-temporal graph reasoning,” in Proceedings of the IEEE/CVF International Conference on Computer Vision (Seoul: IEEE), 5724–5733.

[B14] Fragkiadaki K. Levine S. Felsen P. Malik J. (2015). “Recurrent network models for human dynamics,” in Proceedings of the IEEE International Conference on Computer Vision (Santiago: IEEE), 4346–4354.

[B15] Gao Z. Guo L. Guan W. Liu A.-A. Ren T. Chen S. (2020). A pairwise attentive adversarial spatiotemporal network for cross-domain few-shot action recognition-r2. IEEE Trans. Image Process. 30, 767–782. 10.1109/TIP.2020.3038372 33232234

[B16] Ge F. Hu J. Zhu Y.-H. Arif M. Yu D.-J. (2022a). TargetMM: accurate missense mutation prediction by utilizing local and global sequence information with classifier ensemble. Combin. Chem. High Throughput Screen. 25, 38–52. 10.2174/1386207323666201204140438 33280588

[B17] Ge F. Zhang Y. Xu J. Muhammad A. Song J. Yu D.-J. (2022b). Prediction of disease-associated nsSNPs by integrating multi-scale resnet models with deep feature fusion. Brief. Bioinform. 23, bbab530. 10.1093/bib/bbab530 34953462 PMC8769912

[B18] Ge F. Zhu Y.-H. Xu J. Muhammad A. Song J. Yu D.-J. (2021). MutTMpredictor: robust and accurate cascade XGBoost classifier for prediction of mutations in transmembrane proteins. Comput. Struct. Biotechnol. J. 19, 6400–6416. 10.1016/j.csbj.2021.11.024 34938415 PMC8649221

[B19] Ge S. Zhao S. Gao X. Li J. (2019). “Fewer-shots and lower-resolutions: towards ultrafast face recognition in the wild,” in Proceedings of the 27th ACM International Conference on Multimedia, 229–237.

[B20] Gui L.-Y. Wang Y.-X. Liang X. Moura J. M. (2018). “Adversarial geometry-aware human motion prediction,” in Proceedings of the European Conference on Computer Vision (ECCV) (Munich), 786–803.

[B21] Guo X. Choi J. (2019). “Human motion prediction via learning local structure representations and temporal dependencies,” in Proceedings of the AAAI Conference on Artificial Intelligence, 2580–2587.27534393

[B22] Iida S. Kimura R. Cui H. Hung P.-H. Utsuro T. Nagata M. (2019). “A multi-hop attention for rnn based neural machine translation,” in Proceedings of The 8th Workshop on Patent and Scientific Literature Translation, 24–31.

[B23] Ionescu C. Papava D. Olaru V. Sminchisescu C. (2013). Human3. 6m: large scale datasets and predictive methods for 3D human sensing in natural environments. IEEE Trans. Pattern Anal. Mach. Intell. 36, 1325–1339. 10.1109/TPAMI.2013.248 26353306

[B24] Jain A. Zamir A. Savarese S. Saxena A. (2016a). “Deep learning on spatio-temporal graphs,” in Proceedings of the IEEE Conference on Computer Vision and Pattern Recognition (Las Vegas, NV), 27–30.

[B25] Jain A. Zamir A. R. Savarese S. Saxena A. (2016b). “Structural-RNN: deep learning on spatio-temporal graphs,” in Proceedings of the IEEE Conference on Computer Vision and Pattern Recognition (Seattle, WA: IEEE), 5308–5317.

[B26] Lebailly T. Kiciroglu S. Salzmann M. Fua P. Wang W. (2020). “Motion prediction using temporal inception module,” in Proceedings of the Asian Conference on Computer Vision.

[B27] Li C. Zhang Z. Lee W. S. Lee G. H. (2018). “Convolutional sequence to sequence model for human dynamics,” in Proceedings of the IEEE Conference on Computer Vision and Pattern Recognition, 5226–5234.

[B28] Li M. Chen S. Zhang Y. Tsang I. (2020a). “Graph cross networks with vertex infomax pooling,” Advances in Neural Information Processing Systems, arXiv [Preprint]. arXiv: 2010.01804.

[B29] Li M. Chen S. Zhao Y. Zhang Y. Wang Y. Tian Q. (2020b). “Dynamic multiscale graph neural networks for 3D skeleton based human motion prediction,” in Proceedings of the IEEE/CVF Conference on Computer Vision and Pattern Recognition, 214–223.

[B30] Liu H. Wang L. (2017). Human motion prediction for human-robot collaboration. J. Manufact. Syst. 44, 287–294. 10.1016/j.jmsy.2017.04.009

[B31] Liu Q. Liu Z. Xiong B. Xu W. Liu Y. (2021). Deep reinforcement learning-based safe interaction for industrial human-robot collaboration using intrinsic reward function. Adv. Eng. Inform. 49, 101360. 10.1016/j.aei.2021.101360

[B32] Liu R. Liu C. (2020). Human motion prediction using adaptable recurrent neural networks and inverse kinematics. IEEE Control Syst. Lett. 5, 1651–1656. 10.1109/LCSYS.2020.3042609

[B33] Liu Z. Liu Q. Xu W. Liu Z. Zhou Z. Chen J. (2019a). Deep learning-based human motion prediction considering context awareness for human-robot collaboration in manufacturing. Proc. CIRP 83, 272–278. 10.1016/j.procir.2019.04.080

[B34] Liu Z. Wu S. Jin S. Liu Q. Lu S. Zimmermann R.. (2019b). “Towards natural and accurate future motion prediction of humans and animals,” in Proceedings of the IEEE/CVF Conference on Computer Vision and Pattern Recognition (Long Beach, CA: IEEE), 10004–10012.

[B35] Ma T. Nie Y. Long C. Zhang Q. Li G. (2022). “Progressively generating better initial guesses towards next stages for high-quality human motion prediction,” in Proceedings of the IEEE/CVF Conference on Computer Vision and Pattern Recognition, 6437–6446.

[B36] Mao W. Liu M. Salzmann M. Li H. (2019). “Learning trajectory dependencies for human motion prediction,” in Proceedings of the IEEE/CVF International Conference on Computer Vision (Seoul: IEEE), 9489–9497.

[B37] Mao W. Liu M. Salzmann M. Li H. (2021). Multi-level motion attention for human motion prediction. Int. J. Comput. Vis. 129, 2513–2535. 10.1007/s11263-021-01483-7

[B38] Martinez J. Black M. J. Romero J. (2017). “On human motion prediction using recurrent neural networks,” in Proceedings of the IEEE Conference on Computer Vision and Pattern Recognition (Honolulu, HI: IEEE), 2891–2900.

[B39] Oguz O. S. Gabler V. Huber G. Zhou Z. Wollherr D. (2017). “Hybrid human motion prediction for action selection within human-robot collaboration,” in International Symposium on Experimental Robotics (Tokyo: Springer), 289–298.

[B40] Sang H.-F. Chen Z.-Z. He D.-K. (2020). Human motion prediction based on attention mechanism. Multimedia Tools Appl. 79, 5529–5544. 10.1007/s11042-019-08269-7

[B41] Shan W. Liu Z. Zhang X. Wang S. Ma S. Gao W. (2022). P-STMO: pre-trained spatial temporal many-to-one model for 3d human pose estimation. arXiv preprint arXiv:2203.07628. 10.1007/978-3-031-20065-6_27

[B42] Shu X. Zhang L. Qi G.-J. Liu W. Tang J. (2021). Spatiotemporal co-attention recurrent neural networks for human-skeleton motion prediction. IEEE Trans. Pattern Anal. Mach. Intell. 44, 3300–3315. 10.1109/TPAMI.2021.3050918 33434123

[B43] Sikander R. Arif M. Ghulam A. Worachartcheewan A. Thafar M. A. Habib S. (2022). Identification of the ubiquitin–proteasome pathway domain by hyperparameter optimization based on a 2D convolutional neural network. Front. Genet. 13, 851688. 10.3389/fgene.2022.851688 35937990 PMC9355632

[B44] Tabassum S. Pereira F. S. Fernandes S. Gama J. (2018). Social network analysis: an overview. Wiley Interdiscipl. Rev. Data Mining Knowl. Discovery 8, e1256. 10.1002/widm.1256

[B45] Tang R. Yang G. Wei H. Mao Y. Ture F. Lin J. (2018). Streaming voice query recognition using causal convolutional recurrent neural networks. arXiv preprint arXiv:1812.07754. 10.48550/arXiv.1812.07754

[B46] Tang Y. Ma L. Liu W. Zheng W. (2018). Long-term human motion prediction by modeling motion context and enhancing motion dynamic. arXiv preprint arXiv:1805.02513. 10.48550/arXiv.1805.02513

[B47] Wang R. Panju M. Gohari M. (2017). Classification-based RNN machine translation using GRUs. arXiv preprint arXiv:1703.07841. 10.48550/arXiv.1703.07841

[B48] Yao J. Zhang J. Li J. Zhuo L. (2021). Anchor voiceprint recognition in live streaming via RawNet-SA and gated recurrent unit. EURASIP J. Audio Speech Music Process. 2021, 1–18. 10.1186/s13636-021-00234-3

