# Corrigendum: An initial prediction and fine-tuning model based on improving GCN for 3D human motion prediction

**DOI:** 10.3389/fncom.2023.1232765

**Published:** 2023-06-13

**Authors:** Zhiquan He, Lujun Zhang, Hengyou Wang

**Affiliations:** ^1^Guangdong Key Laboratory of Intelligent Information Processing, Shenzhen, China; ^2^Guangdong Multimedia Information Service Engineering Technology Research Center, Shenzhen University, Shenzhen, China; ^3^School of Science, Beijing University of Civil Engineering and Architecture, Beijing, China

**Keywords:** motion prediction, GCN-based, two-stage prediction method, spatial attention, causally temporal

In the published article, there was an error in the **Funding** statement. The Shenzhen City project support was omitted. The correct **Funding** statement appears below.

